# Limitations to usual activities due to health problems in Germany

**DOI:** 10.17886/RKI-GBE-2017-071

**Published:** 2017-10-09

**Authors:** Elena von der Lippe, Angela Fehr, Cornelia Lange

**Affiliations:** Robert Koch Institute, Department of Epidemiology and Health Monitoring, Berlin

**Keywords:** GLOBAL ACTIVITY LIMITATION INDICATOR (GALI), HEALTHY LIFE YEARS, HEALTH MONITORING, GERMANY

## Abstract

Limitations to usual activities due to health problems impact quality of life and well-being. The Global Activity Limitation Indicator (GALI) was developed to assess the trends in limitations to usual activities. The GALI question is applied in a single or routed, multi-question version. GEDA 2014/2015-EHIS for the first time applied a three-question version. Three quarters of respondents reported that they did not experience any limitations to their usual activities. 18.7% of women and 17.0% of men said they had experienced moderate limitations. 6.4% of women and 6.8% of men reported severe limitations. The share of respondents experiencing limitations increases with age and shows a clear education gradient. Changes in methodology, however, mean that current results based on the GALI question cannot be compared to the results from earlier survey waves of the same study.

## Introduction

Physical or mental health problems that lead to limitations in usual activities have the potential to significantly impact quality of life and personal well-being. Given the demographic change and the rise in life expectancy, the number of people experiencing limitations to their usual activities will likely increase in the future. The years of life gained may be spent in good or bad health [1, 2]. A key challenge for health policy, therefore, will be to provide the conditions for people to age in good health and maintain their quality of life. That is why it is crucial to investigate if the rise in life expectancy is related to an increase in years spent without limitations to usual activities due to health problems. There seems to be a trend in Germany towards the occurrence of diseases and limitations to usual activities at a later stage in life and therefore towards a longer life with good health [3, 4].

Political measures and preventive healthcare programmes that focus on these developments require an indicator that determines the share of people in the population affected by limitations. The Global Activity Limitation Indicator (GALI) was developed to measure such trends in the limitations to usual activities over time. The GALI question collects data on self-rated limitations. Data obtained through the GALI question is used to calculate the Healthy Life Years (HLY) indicator, which is also known as disability free life expectancy. It provides information on the number of remaining life years that respondents will spend without limitations to usual activities due to health problems [5]. The GALI question is part of the Minimum European Health Module (MEHM) [6] and is also surveyed in the context of the EU-Statistics on Income and Living Conditions (EU-SILC). Prütz and Lange 2016 provide an overview of the available data sources on disability and social participation [7]. Issue 1/2017 of the Journal of Health Monitoring contains an overview of European health indicators [8].

**GEDA 2014/2015-EHIS**

**Data holder:** Robert Koch Institute

**Aims:** To provide reliable information about the population’s health status, health-related behaviour and health care in Germany, with the possibility of a European comparison

**Method:** Questionnaires completed on paper or online

**Population:** People aged 18 years and above with permanent residency in Germany

**Sampling:** Registry office sample; randomly selected individuals from 301 communities in Germany were invited to participate

**Participants:** 24,016 people (13,144 women; 10,872 men)

**Response rate:** 26.9%

**Study period:** November 2014 - July 2015

**Data protection:** This study was undertaken in strict accordance with the data protection regulations set out in the German Federal Data Protection Act and was approved by the German Federal Commissioner for Data Protection and Freedom of Information. Participation in the study was voluntary. The participants were fully informed about the study’s aims and content, and about data protection. All participants provided written informed consent.

More information in German is available at

www.geda-studie.de

## Indicator

The prevalence of limitations to usual activities was surveyed in GEDA 2014/2015-EHIS through self-administered paper-based or online questionnaires. Respondents were asked: ‘Are you limited because of a health problem in activities people usually do? (Yes/No)’ Respondents who answered with yes were then asked two further questions: a) ‘How severely are you limited in your usual activities? (Severely limited/Moderately limited)’; b) ‘For how long have you been limited? (For less than six months/For six months or longer)’. These responses provide the basis to form three categories: severely limited, moderately limited and not limited. Respondents who answered that they were moderately or severely limited in their usual activities for over 6 months are considered as limited due to health problems. If not clarified otherwise, the term limitations in the following refers to the collapsed group of moderately and severely limited.

The analyses are based on data from 23,752 participants aged 18 and above (13,014 women and 10,738 men) with valid data on the GALI question. The calculations were carried out using a weighting factor that corrects for deviations within the sample from the German population (as of 31 December 2014) with regard to gender, age, district type and education. The district type reflects the degree of urbanisation and accounts for the regional distribution in Germany. The International Standard Classification of Education (ISCED) was used to classify the responses provided on educational level [9]. Differences between these groups are interpreted as statistically significant if the respective confidence intervals do not overlap.

A detailed description of the methodology applied in the GEDA 2014/2015-EHIS study can be found in Lange et al. 2017 [10] as well as in the article German Health Update: New data for Germany and Europe in Issue 1/2017 of the Journal of Health Monitoring.

## Results and discussion

Three quarters of respondents reported no limitations to their usual activities. 25.2% of women and 23.8% of men said they are long-term limited in their usual activities due to health problems (Table 1 and Table 2). 6.4% of women and 6.8% of men reported severe limitations to their usual activities. When looking at both categories together, severely and moderately limited, it becomes evident that the share of respondents experiencing limitations strongly increases with age. Whereas 12.0% of women and 9.5% of men aged 18 to 29 reported limitations, this figure rises to 41.1% of women and 39.4% of men aged 65 and over. A clear education gradient is evident for both sexes regarding limitations. Across all age groups, the share of women and men facing limitations to usual activities due to health problems is significantly lower for those with high education than those with low education.

**Figure 1:**
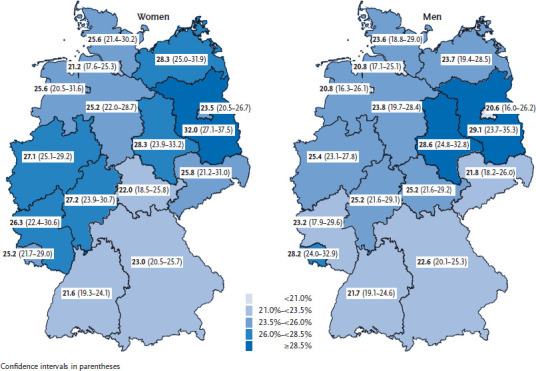
12-month prevalence of limitations due to health problems (severely and moderately limited) according to gender and federal state (n=13,014 women; n=10,738 men) Source: GEDA 2014/2015-EHIS

Regional differences in the share of people experiencing limitations also exist (Figure 1). Compared to the German average, prevalence among women from Baden-Württemberg (21.6%) is significantly lower, while among women from Brandenburg it is significantly higher (32.0%). For men, no such differences compared to the German average were observed. A significant difference was recorded for men from Baden-Württemberg (21.7%) compared to men from Saxony-Anhalt (28.6%). These results, however, do not take into account the regional differences in the age composition of the population. The different age patterns in the federal states could explain regional differences regarding limitations due to health problems. Studies show that the shift between the age groups is stronger in East than in West Germany. In East Germany, the share of those aged 65 and over is 23%, whereas in the west it is 21% [11].

The GALI question can be implemented in different ways. Whereas GEDA 2014/2015-EHIS applied the three-question version (see above), data for the indicator can also be collected through a single question with multiple dimensions (existence of a limitation, its duration and severity). Previous GEDA survey waves [12-14] used a single-question version. In 2015, the German questionnaire for the EU-Statistics on Income and Living Conditions (EU-SILC), implemented in Germany by the Federal Statistical Office, applied the three-question version, thereby deviating from earlier surveys. The change in the formulation of the question means that current results for the GALI question are not comparable to the results from previous survey waves of the same study. This applies both to the GEDA survey and to EU-SILC. However, similar shifts in the results of both surveys compared to their earlier waves are observed. In GEDA 2014/2015-EHIS, 75.5% of respondents report that they do not experience limitations, whereas in GEDA 2012 the corresponding figure was 67.1%. The share of respondents who experience severe or moderate limitations (GEDA 2012) changed between GEDA 2012 (11.3%) and GEDA 2014/2015-EHIS (6.6%) as did the share of those experiencing moderate limitations (GEDA 2012 21.7%; GEDA 2014/2015-EHIS 17.9%). Published EU-SILC results for Germany reveal that in 2015 21.2% of the population experienced limitations to their usual activities (women 21.7%; men 20.6%) [15]. In comparison, in 2014, 36.2% of respondents reported limitations to usual activities [16]. Independently of each other, 2015 EU-SILC and GEDA 2014/2015-EHIS data therefore reveal similar shifts to previous survey waves. These results highlight that the indicator is sensitive to changes in the way questions are posed [17]. This aspect will be further analysed and discussed in order to identify relevant potentials for improvement.

The demographic change and the rise in life expectancy will require changes at the level of prevention and healthcare provision. Periodically collected data on limitations to usual activities due to health problems and the extent and duration of such limitations are a potentially valuable contribution to strengthening needs-based prevention and healthcare, also at regional level, where applicable.

## Key statements

- Three quarters of GEDA 2014/2015-EHIS respondents report no limitations to usual activities.
- 6.4% of women and 6.8% of men report they are severely limited.
- The share of respondents reporting limitations increases with age and shows a strong education gradient.
- There are some significant differences in limitations to usual activities between federal states.

## Figures and Tables

**Table 1:** 12-month prevalence of limitations due to health problems among women according to age and educational level (n=13,014 women) Source: GEDA 2014/2015-EHIS

Women	Not limited		Moderately limited		Severely limited		Limited (moderately and severely limited)	
	%	(95% CI)	%	(95% CI)	%	(95% CI)	%	(95% CI)
**Women total**	**74.8**	**(73.9-75.8)**	**18.7**	**(17.9-19.6)**	**6.4**	**(5.8-7.1)**	**25.2**	**(24.2-26.1)**
**18-29 Years**	88.0	(86.2-89.6)	9.9	(8.5-11.6)	2.0	(1.3-3.1)	12.0	(10.4-13.8)
Low education	82.3	(76.8-86.7)	12.1	(8.6-16.7)	5.6	(3.2-9.6)	17.7	(13.3-23.2)
Medium education	89.2	(87.1-91.0)	9.7	(8.0-11.7)	1.1	(0.5-2.2)	10.8	(9.0-12.9)
High education	92.5	(89.1-94.9)	7.1	(4.8-10.4)	0.4	(0.1-1.5)	7.5	(5.1-10.9)
**30-44 Years**	86.4	(84.6-87.9)	11.6	(10.3-13.1)	2.0	(1.5-2.8)	13.6	(12.1-15.4)
Low education	82.9	(77.0-87.5)	12.0	(8.4-16.9)	5.1	(2.8-8.9)	17.1	(12.5-23.0)
Medium education	84.9	(82.7-86.8)	13.3	(11.5-15.3)	1.8	(1.2-2.8)	15.1	(13.2-17.3)
High education	91.7	(89.8-93.2)	7.5	(6.0-9.4)	0.8	(0.4-1.6)	8.3	(6.8-10.2)
**45-64 Years**	73.7	(72.0-75.4)	20.5	(19.0-22.1)	5.8	(5.0-6.6)	26.3	(24.6-28.0)
Low education	64.7	(59.8-69.3)	24.2	(20.4-28.6)	11.1	(8.4-14.5)	35.3	(30.7-40.2)
Medium education	74.5	(72.4-76.4)	20.2	(18.4-22.1)	5.3	(4.5-6.4)	25.5	(23.6-27.6)
High education	79.4	(76.9-81.7)	18.1	(16.0-20.5)	2.5	(1.7-3.6)	20.6	(18.3-23.1)
**≥ 65 Years**	58.9	(56.7-61.1)	27.6	(25.6-29.6)	13.5	(11.9-15.3)	41.1	(38.9-43.3)
Low education	54.2	(50.6-57.9)	29.1	(25.9-32.5)	16.6	(14.1-19.5)	45.8	(42.1-49.4)
Medium education	61.6	(58.5-64.6)	26.4	(24.0-28.9)	12.0	(9.9-14.5)	38.4	(35.4-41.5)
High education	65.7	(60.3-70.7)	26.5	(21.9-31.7)	7.8	(5.4-11.1)	34.3	(29.3-39.7)
**Total (women and men)**	**75.5**	**(74.7-76.3)**	**17.9**	**(17.2-18.5)**	**6.6**	**(6.2-7.1)**	**24.5**	**(23.7-25.3)**

CI=Confidence interval

**Table 2:** 12-month prevalence of limitations due to health problems among men according to age and educational level (n=10,738 men) Source: GEDA 2014/2015-EHIS

Men	Not limited		Moderately limited		Severely limited		Limited (moderately and severely limited)	
	%	(95% CI)	%	(95% CI)	%	(95% CI)	%	(95% CI)
**Men total**	**76.2**	**(75.1-77.2)**	**17.0**	**(16.1-17.9)**	**6.8**	**(6.2-7.5)**	**23.8**	**(22.8-24.9)**
**18-29 Years**	90.5	(88.6-92.2)	7.0	(5.6-8.8)	2.5	(1.6-3.7)	9.5	(7.8-11.4)
Low education	87.6	(82.8-91.2)	6.8	(4.3-10.5)	5.6	(3.3-9.5)	12.4	(8.8-17.2)
Medium education	91.1	(88.6-93.1)	7.6	(5.7-9.9)	1.3	(0.6-2.8)	8.9	(6.9-11.4)
High education	93.4	(88.5-96.3)	5.2	(3.1-8.7)	1.4	(0.2-9.2)	6.6	(3.7-11.5)
**30-44 Years**	85.9	(83.9-87.8)	11.4	(9.8-13.2)	2.6	(1.8-3.8)	14.1	(12.2-16.1)
Low education	77.2	(68.9-83.8)	14.7	(9.5-22.2)	8.1	(4.4-14.3)	22.8	(16.2-31.1)
Medium education	84.2	(81.2-86.8)	13.3	(10.9-16.1)	2.5	(1.6-3.9)	15.8	(13.2-18.8)
High education	92.5	(90.4-94.1)	6.9	(5.3-8.9)	0.6	(0.3-1.5)	7.5	(5.9-9.6)
**45-64 Years**	72.5	(70.6-74.3)	19.6	(18.1-21.3)	7.9	(6.9-9.1)	27.5	(25.7-29.4)
Low education	65.9	(60.7-70.8)	22.0	(18.2-26.3)	12.1	(8.9-16.1)	34.1	(29.2-39.3)
Medium education	67.9	(65.0-70.6)	22.5	(20.1-25.1)	9.7	(8.2-11.4)	32.1	(29.4-35.0)
High education	83.2	(81.2-85.1)	13.6	(11.9-15.5)	3.2	(2.3-4.3)	16.8	(14.9-18.8)
**≥ 65 Years**	60.6	(58.5-62.8)	26.4	(24.5-28.4)	13.0	(11.6-14.6)	39.4	(37.2-41.5)
Low education	57.7	(52.8-62.6)	27.3	(22.9-32.1)	15.0	(12.0-18.6)	42.3	(37.4-47.2)
Medium education	60.2	(56.9-63.4)	25.7	(23.0-28.6)	14.1	(12.0-16.5)	39.8	(36.6-43.1)
High education	62.6	(59.2-65.9)	27.3	(24.0-31.0)	10.1	(8.0-12.5)	37.4	(34.1-40.8)
**Total (women and men)**	**75.5**	**(74.7-76.3)**	**17.9**	**(17.2-18.5)**	**6.6**	**(6.2-7.1)**	**24.5**	**(23.7-25.3)**

CI=Confidence interval
